# In silico methods for predicting functional synonymous variants

**DOI:** 10.1186/s13059-023-02966-1

**Published:** 2023-05-22

**Authors:** Brian C. Lin, Upendra Katneni, Katarzyna I. Jankowska, Douglas Meyer, Chava Kimchi-Sarfaty

**Affiliations:** Hemostasis Branch 1, Division of Hemostasis, Office of Plasma Protein Therapeutics CMC, Office of Therapeutic Products, Center for Biologics Evaluation and Research, US FDA, Silver Spring, MD USA

## Abstract

Single nucleotide variants (SNVs) contribute to human genomic diversity. Synonymous SNVs are previously considered to be “silent,” but mounting evidence has revealed that these variants can cause RNA and protein changes and are implicated in over 85 human diseases and cancers. Recent improvements in computational platforms have led to the development of numerous machine-learning tools, which can be used to advance synonymous SNV research. In this review, we discuss tools that should be used to investigate synonymous variants. We provide supportive examples from seminal studies that demonstrate how these tools have driven new discoveries of functional synonymous SNVs.

**Supplementary Information:**

The online version contains supplementary material available at 10.1186/s13059-023-02966-1.

## Background

The primary source for evolutionary diversity is genetic variation [1, 2]. Single nucleotide variants (SNVs) make up only ~ 0.1% of the entire human genome but are responsible for differences in the human population, including disease susceptibility and response to drugs [3]. SNVs can be divided into nonsynonymous variants, which alter the encoded amino acids, or synonymous variants that alter the codon sequence, but preserve the native amino acid structure. While the effects of nonsynonymous variants are evident, synonymous variants have been assumed to be neutral and yield minimal functional consequences. Compelling evidence over the last decade has disputed this view, and both in silico and experimental studies have revealed a variety of effects of synonymous variants, spanning from alterations to RNA structure to changes in protein expression and function to engendering adaptive evolution [4–7]. In fact, synonymous variants have now been implicated in cancers [8] and over 85 genetic diseases [9] and are responsible for many cellular disruptions at both the RNA and protein levels [7, 10]. The most prominent effects include changes to RNA structure/stability [11], splicing [12, 13], and miRNA binding [14, 15]. As these mechanisms mostly result from direct changes to the nucleotide sequence, in silico tools have been applied in both the discovery of pathogenic synonymous variants and in their characterization [16, 17]. To date, many notable studies on synonymous variants have implemented a dual strategy: first, using in silico tools to screen and predict for functional variants, and second, applying sensitive experimental techniques to validate these in silico predictions [7, 9, 11, 18–20]. Undoubtedly, the rising incorporation of computational approaches in biological research has driven a significant increase in discoveries of functional and pathogenic synonymous variants [21]. Though still in its infancy, many in silico variant predictors represent promising methods to distinguish between pathogenic and benign synonymous variants [22–24].

In addition, the computational field has also undergone a significant transformation. Through machine-learning (ML) and deep learning (DL) platforms, in silico tools have evolved to better integrate biological factors and experimental data into their algorithms [25]. Many tools use publicly available genetic datasets to train the ML systems to better predict functional variants [26–28]. New tools continue to be developed with unprecedented improvements in predictability and accuracy, and in many cases, substantial updates have been released, which have refined many popular existing tools. As researchers continue to acknowledge the importance of sequence properties, such as codon usage and GC content, in determining protein characteristics and new metrics and resources have been adopted for their evaluation [29–33], these dimensions have further enriched prediction models. Currently, well over a hundred tools have been used to characterize variants, each with their own specific predictive algorithms, but also with limitations that must be accounted for. While in silico tools have advanced research, their rapid development has also posed a conundrum of whether a single tool is preeminent or if multiple tools should be used. To realize the full potential of these in silico tools in synonymous variant research, further integration of these tools into a consistent workflow and substantiation of the predicted results through experimental data are required.

**Fig. 1:**
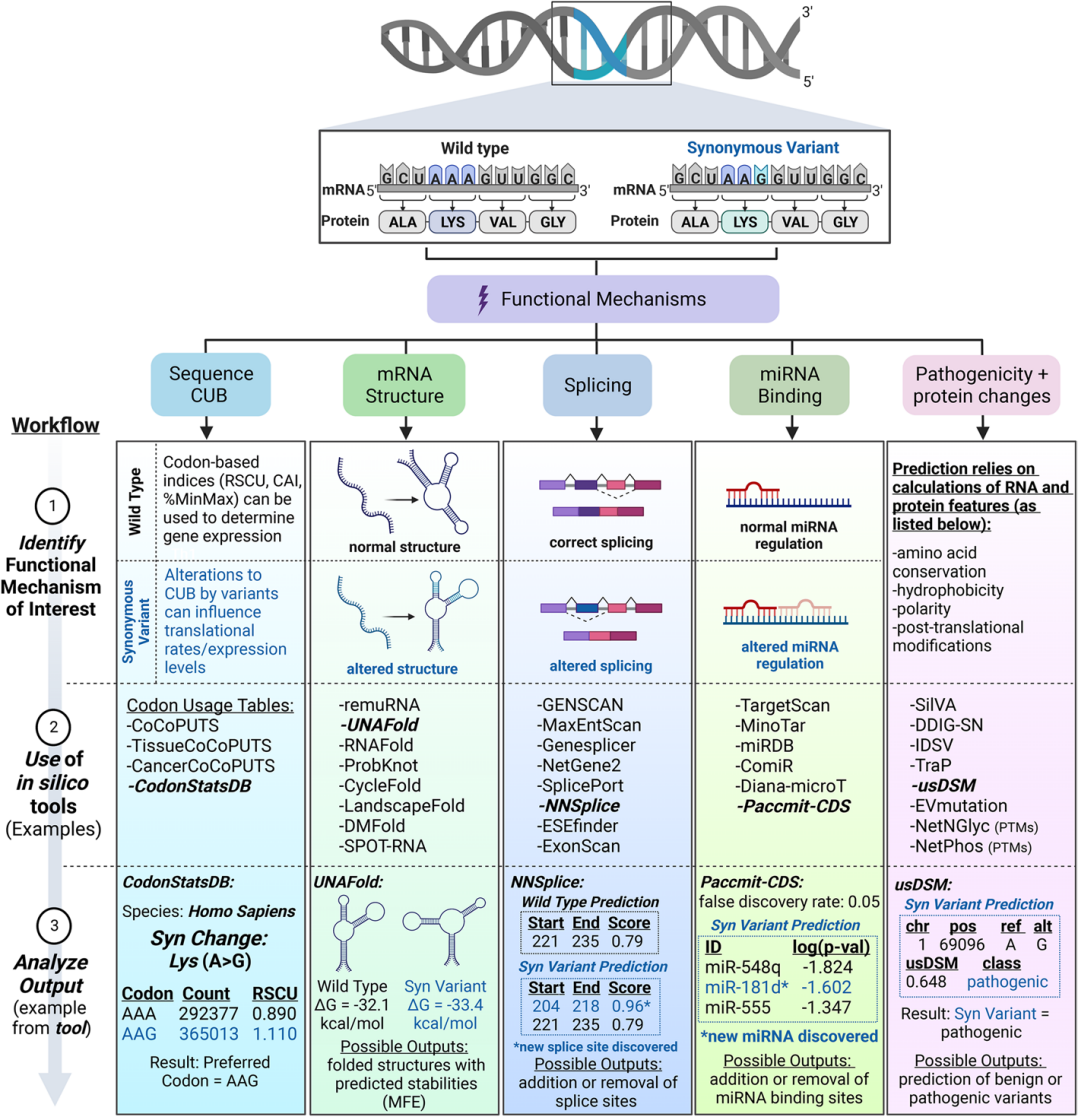
Workflow schematic for how to optimally use in silico tools to investigate synonymous variants. Genetic sequences containing synonymous variants can cause many different functional effects, including alterations to codon usage biases, mRNA structure, splicing, miRNA binding, disease pathogenesis, and protein characteristics. After (1) identifying a functional mechanism of interest, (2) a variety of different in silico tools can be chosen and applied to evaluate the sequence containing synonymous variants. After the sequence has been processed, (3) outputs of these tools can be analyzed to form predictions. For proper evaluation, most tools will require input of a short nucleotide sequence containing the synonymous variant. The wild-type sequence for the identical region encompassing the synonymous variant should be processed for comparison. Examples of potential outputs for tools highlighted in row 2 are shown in row 3. CodonStatsDB determines codon preferences based on RSCU values. UNAFold can generate predicted mRNA structures and calculate differences in mRNA stability. NNSplice will reveal any new or lost splice sites. Paccmit-CDS is able to capture changes to miRNA binding sites. usDSM is able to predict the pathogenicity of the variant. Outputs may vary depending on the algorithms and structure of the tools. It is highly beneficial to analyze the sequence through multiple tools and to validate the results through experimental methods

In this review, we highlight the process by which in silico tools should be used to effectively characterize synonymous variants (Fig. 1), while providing numerous examples from studies that have successfully implemented these methods. We characterize the differences among in silico tools by sorting them into sections based on their intended functions and provide a framework for how these tools should be optimally used to investigate various effects of synonymous variants. This review will discuss the most commonly utilized tools and introduce many that were more recently developed to provide a thorough resource for applying in silico tools in the study of synonymous variants.

## In silico resources for assessing codon usage and sequence properties of synonymous variants

Genomes of most organisms are degenerate with multiple different codons translated into the same amino acid. However, synonymous codons are not used in a uniform fashion and genomes are biased to favor particular codons. Sharp and Li characterized this codon usage bias (CUB) in *Escherichia coli and Salmonella typhimurium* genes by introducing two metrics, Codon Adaptation Index (CAI) and Relative Synonymous Codon Usage (RSCU) [34]. Around the same time, another popular measure of CUB was devised called the expected number of codons (ENC), quantifying how far a gene’s codon usage deviates from equal usage of synonymous codons [35]. These metrics formed the original systems to score gene level CUB, computing the difference between scores assigned to wildtype sequences and sequences containing synonymous variants.

Today, while these methods continue to be used extensively, new insights into translational processes have led to the creation of additional methods to quantify CUB. Commonly used codons are thought to correlate with more abundant tRNAs [36–38], leading to the development of the tRNA Adaptation Index (tAI) based on tRNA usages [39] and a species-specific tAI calculator (stAI_calc_) that infers organism-specific tAI wobble weights for 100 different species [40]. In addition, non-random codon biases have been found to impact translation kinetics and co-translational folding [31–33, 41–44]. Moura et al. reported that both missense and synonymous mutations are under selective pressure to maintain usage of codon multiples in bacteria, archaea, and eukaryotes [45]. Codon pairs, two adjacent codons (i.e., bicodon), also exhibit usage biases that have been found to impact translational efficiencies [46]. Others have reported that codon pair frequencies provide no additional information towards predicting expression than single codon frequencies in S. cerevisiae [47] and that viral codon pair usage bias is dictated primarily by avoiding certain dinucleotides [48]. By distinguishing rare or optimal codons, many metrics can be used to identify synonymous variants that impact protein properties through disrupting translational kinetics and co-translational folding [49]. For this purpose, Rodriguez et al. developed the %MinMax tool to calculate synonymous codon usage with a focus on measuring deviations in optimal cotranslational folding patterns [29].

Furthermore, in multicellular organisms, CUB can vary across different tissue contexts. Plotkin et al. reported tissue-specific codon usage patterns by comparing groups of human genes previously reported to be expressed in specific tissues [50]. Similarly, Qingpo Liu found differences in codon usage between tissue-specific genes in rice [51]. tRNA expression differs among human tissues [52]. Therefore, CUB metrics should incorporate tissue-specific contexts into its calculations. In recent years, two databases have been assembled to aid in these tissue-specific calculations: TissueCoCoPUTs, which uses transcriptomic data from different tissue contexts to compute a weighted average codon usage in several different tissue contexts [32] and CancerCoCoPUTs, which reports differences in codon usage across several different solid tumor types [33]. These resources, along with large databases, such as the Codon Statistics Database [53], have made it remarkably effortless to evaluate CUB and sequence properties of synonymous variants.

## In silico tools for assessing the effect of synonymous variants on mRNA structure and stability

Synonymous variants can have functional and disease consequences through altering mRNA secondary structure and stability. Encoded within the primary mRNA sequence is the information to establish local mRNA secondary structure motifs and dictate RNA stability of individual regions, which can determine the accessibility of ribosome binding sites and speed of local translation [54–56]. One seminal discovery in the field of synonymous variants was the observation that in the mutated *CFTR* gene (c.1520_1522delTCT), which causes cystic fibrosis, a single synonymous variant (c.507 T > A) [18, 57, 58] caused the formation of two enlarged loops in the mRNA structure [18]. This deviation correlated with a reduction in translational rate and reduced expression of the CFTR protein [18]. While this finding was validated experimentally through RNA folding assays and circular dichroism analysis, like many other studies, its initial discovery was uncovered through molecular modeling.

In essence, RNA structure and its folding process have been found to be deeply rooted in a couple of principles, which has inspired the development of RNA structure prediction tools. First, RNA secondary structure evolutionarily favors stability, except for select situations where unstable areas in the transcript, such as at the 5′ end, supports translational initiation [59–61]. Stable RNA provides many benefits, including increased half-life, fine-tuning of translational speed, and establishing favorable binding sites for RNA-binding proteins and miRNAs [62, 63]. mRNA conforms to structures that more easily maintain its structural integrity, which in most cases, the realized structure is one that possesses the lowest free energy [64, 65]. However, although a single structure may be the most stable and dominant, multiple structures co-exist within the dynamic cellular environment. RNA populates a heterogeneous ensemble of conformations, and the goal of most prediction tools is to differentiate the native structure from its numerous subpopulations [66]. Second, across species, coding regions contain many structurally conserved elements [59, 67–69], which can be used to infer both function and structure. Based on these assumptions, many tools have been established with algorithms designed to identify the minimum free energy (MFE) structure with consideration of conserved motifs, temperature, ion concentrations, and sequence-based properties.

In silico tools, such as mFold [70] (recently updated and renamed to UNAFold [71]), remuRNA [72], Kinefold [73], CoFold [74], and RNAfold [75], are examples of tools that predict structures based on algorithms to minimize free energy. These tools require input of RNA sequences with recommended length limit of < 1500 nucleotides as longer sequences significantly increase folding complexities and software run-time. These tools are extensively used to generate predicted mRNA structures due to their reputable accuracy and fast computing speed. For example, mFold was used in the CFTR study to reveal structural loop elements in the mutated CFTR structure [18]. Likewise, Duan and colleagues [11] used mFold to show that one synonymous mutation (c.957C > T) in human *DRD2* (dopamine receptor D2) led to decreased mRNA stability and decreased expression. In a separate study, mFold, Kinefold and NUPACK [76] were used collectively by Simhadri and colleagues to highlight how a *F9* (Factor IX) synonymous variant (c.459G > A) alters mRNA structure to facilitate changes in protein expression [77].

As applied in these aforementioned studies, prediction tools can be used to simulate folding of both the wild type and mutant sequences and to calculate the free energy of the best candidate structures. A single synonymous variant can perturb the conformational ensemble and shift folding dynamics, thereby forming misfolded or non-native structures of higher or lower free energy (ΔG). Any observed difference in predicted minimum free energies (ΔΔG) between wild type and mutated structures may suggest a change in mRNA structure (example workflow is shown in Fig. 1). The significance of a change in MFE may vary among RNA structures and can be affected by various input parameters. Wayment-Steele and colleagues found that increasing the simulated folding temperature can improve the correlation of predicted structures to experimental data [78]. In addition, sequence length is another factor that can alter the magnitude of MFE differences due to the added complexity of folding larger structures and should be a variable closely considered [16]. Due to these potential factors, these tools provide an effective method to screen for potential RNA structural changes, but results do require further validation through experimental methods.

Additionally, while RNA prediction tools based on MFE are effective at accurately rendering RNA structures that are composed of a high number of canonical Watson–Crick base pairs, RNA folding is dynamic and complex. New insights into the structural topology of RNA has revealed special base pairing configurations, such as pseudoknots and noncanonical intramolecular base pairing patterns that support specific structural contexts (i.e., geometric motifs, higher-order multiplexes) and tertiary interactions [79]. Noncanonical base pairs are base interactions that deviate from the standard Watson–crick base pairings, such as G-A pairs, and pseudoknots are non-nested structures that form from two stem-loops. In consideration of these features, ProbKnot [80], IPKnot [81], Knotty [82], and LandscapeFold [83] are dependable tools used for pseudoknot predictions and MC-Fold-DP [84] and CycleFold [85] are equipped with special features to handle noncanonical base pairs. These are powerful tools that employ sophisticated algorithms to include special base pairings and improve prediction performance but can only consider small nucleotide sequences due to computation times. Nevertheless, shorter sequences can provide significant information about the effects of synonymous variants on mRNA structure, in which subtle changes may occur locally.

New machine-learning approaches are able to circumvent computational time issues because these techniques are data-driven approaches rather than score-dependent. Two ML tools, DMfold [86] and SPOT-RNA [87, 88], have been generated with accuracies that supersede existing tools. These multivariate tools are able to consider free energy parameters, sequence characteristics, and other properties while having the unique advantage of using genetic databases and RNA structure datasets for model training. However, because of their novelty, these ML approaches remain relatively enigmatic, and there remain concerns of potential issues with overfitting and inaccuracies in predicting structures that are more dissimilar to structures that appeared in training sets. Nevertheless, these ML techniques represent the most promising methods for predicting RNA structures and the performance of these tools will likely continue to improve as more publicly available RNA data is collected. Similar to the state of ML RNA prediction tools, computational 3D modeling of complex RNA structures remains a significant challenge but has undergone significant improvements in recent years as more RNA structures have been revealed experimentally and computationally [89]. Eterna (https://eternagame.org/), a crowdsourcing initiative, has rapidly accelerated discoveries in the RNA field and has stimulated improvements in the design of RNA structures for RNA-based therapeutics [78, 90]. Current 3D modeling can be separated into 3 approaches: (i) comparative modeling, in which RNA structures are predicted based on homologous structures (e.g., ModeRNA [91], RNABuilder [92]); (ii) fragment assembly, whereby RNA structures are decomposed into fragments and compared to the target sequence for assembling a predicted structure (e.g., RNAComposer [93], VfoldLA [94]); and (iii) de novo modeling, which relies on coarse grained molecular dynamics and knowledge-based force-field principles to generate structures (e.g., SimRNA [95], iFoldRNA [96]). Many recent reviews and methodology articles provide a thorough overview of the applications of RNA 3D modeling tools [89, 97]. For synonymous variant research, 3D RNA modeling tools have not yet been implemented, but with rapid advancements in this growing field, these tools may be applicable in the near future.

Ultimately, assessing RNA structure with a combination of tools that employ various algorithms and parameters is the most optimal approach to evaluate synonymous variants. Agreement between prediction tools increases confidence in predicted structures, while disagreement suggests that the RNA structure is complex. Recently, computational tools, such as SSRTool [98], have been generated with the goal to distinguish the most likely native structure after assessing predictions from a large class of selected prediction tools. However, when tested against known RNA structures from various different species, the tool was unable to guarantee an optimal structure prediction. Therefore, we recommend the use of multiple tools to evaluate synonymous variants and to complement these in silico studies with experimental approaches. A comprehensive list of tools used for assessing synonymous variants is shown in Table 1.

**Table 1:** In silico tools for predicting effects of synonymous variants on mRNA structure

Prediction Algorithms	Tool	Input	Output	Special features	Notes	URL	Ref
Free energy minimization	CoFold	Single nucleotide sequence: limit of 50 kb; 1500 nt sequence has a run-time of approximately ~ 15 s	Structure diagram + predicted MFE (visualized as an arc plot and supports many other output formats)	Two different thermodynamic parameter options + scaling choices	Algorithm considers co-transcriptional folding to improve accuracy of predicting structure of longer sequences	https://e-rna.org/cofold/	[74]
	remuRNA	Wild-type and mutant sequence (no upper limit)	Structure diagram + predicted MFE; relative entropy plot		Algorithm incorporates relative entropy between Boltzmann ensembles of wild-type and mutant secondary structures	https://github.com/bgruening/galaxytools/tree/master/tools/rna_tools/remurna	[72]
	RNAfold	Single nucleotide sequence: 7500 nt limit for partition function calculations; 10,000 nt limit for free energy minimization prediction	Interactive RNA secondary structure plot; mountain plot	Parameter options to avoid isolated base pairs, to use partition function, and/or exclude GU pairs at end of helices	Algorithm employs partition function calculations in addition to free energy minimization	http://rna.tbi.univie.ac.at/cgi-bin/RNAWebSuite/RNAfold.cgi	[75]
	UNAFold	Single nucleotide sequence or multiple short sequences (no upper limit)	Predicted MFE; circular structure plot; energy dot plot	Parameter constraint options to optimize loop types, base numbering frequencies, regularization angles	Uses free energy minimization program with folding temperature fixed at 37 °C	http://www.unafold.org/	[71]
Pseudoknots	IPKnot	Single or multiple sequences (FASTA format or multiple sequence alignments)	2D diagram using VARNA program and structure as an arc plot	Options between multiple scoring models and prediction complexity levels	Uses integer programming to compute the maximum expected accuracy structure (MEA)	http://rtips.dna.bio.keio.ac.jp/ipknot/	[81]
	Kinefold	Single sequence (no upper limit)	Lowest free energy structure diagram + predicted MFE; folding path movie; helix tracing graph	Stochastic simulation—co-transcriptional folding or renaturation folding	Stochastic folding simulations using folding dynamic algorithms [99] and physical constraint modeling for pseudoknot prediction	http://kinefold.curie.fr/	[73]
	Knotty	Single sequence (no upper limit)	Structure diagram + predicted MFE; provides information on all candidate structures		Predicts complex pseudoknot structures with optimization of run-time through sparsification technique and a CCJ-type algorithm	https://github.com/HosnaJabbari/Knotty	[82]
	LandscapeFold	A list of sequences (up to 2), option to consider intramolecular pseudoknots and define minimum number of nucleotides within each hairpin	Identifies all possible structures and provides indexing/sorting via MFE and equilibrium probabilities	Multiple sequence structural analysis for predicting base interactions with option to assess equilibrium concentrations	Polymer physical model based on entropy calculations of arbitrary pseudoknotted structures	https://github.com/ofer-kimchi/RNA-FE-Landscape	[83]
	ProbKnot	Single sequence (no upper limit)	Base pair probability plot	Optimization of iterations and minimum helix length	Predicts for presence of pseudoknots in sequence	https://rna.urmc.rochester.edu/RNAstructureWeb/Servers/ProbKnot/ProbKnot.html	[80]
Noncanonical base pairings	CycleFold	Single or multiple sequences (no upper limit), can apply maximum expected accuracy (MEA) or ProbKnot to generate structures	Lowest MFE structure, matrix of pairing probabilities between each nucleotide sequence	TurboFold mode can be engaged to process multiple sequences, considers evolutionary conservation	Uses nucleotide cyclic motifs to predict noncanonical base pairings and minimizes free energy	http://rna.urmc.rochester.edu	[85]
	MC-Fold-DP	No sequence limit, but runtime scales polynomially	Returns all structures within energy band above the ground state	Cannot currently consider pseudoknots	Prediction based on combining small nucleotide cyclic motifs	https://hackage.haskell.org/package/MC-Fold-DP	[84]
Machine-learning	DMfold	Single sequences (no upper limit)	Folded RNA structure and energy model	Folding parameters automatically determined based on deep learning	Deep-learning and improved base pair maximation principles; trained with 3948 known RNA primary sequences [100]	https://github.com/linyuwangPHD/RNA-Secondary-Structure-Database	[86]
	SPOT-RNA	Single sequence (maximum—2000 nts); can run longer sequences or batch sequences locally	2D plots of structure through VARNA visualization tool, output of secondary structure motifs can be seen through Vienna format		Deep contextual neural network implemented with model training and transfer learning from high quality datasets of > 10,000 RNA structures; trained with bpRNA [101] and PDB [102] databases	https://sparks-lab.org/server/spot-rna/	[87, 88]

## In silico tools for determining effects of synonymous variants on RNA splicing

Pre-mRNA splicing is the co-transcriptional process of excising non-coding introns and joining protein-coding exons. Splicing is mediated by the spliceosome complex, composed of five small nuclear ribonuclear proteins (snRNPs) and more than 150 proteins, and involves recognition of *cis*-acting elements, including 5′ and 3′ splice sites (donor and acceptor sites, respectively), branch point sequences, and polypyrimidine tract (PPT) [103]. A majority of the splice sites (> 98%) have invariant GT and AG as the first and last two intronic nucleotides, respectively, and less conserved sequences in the remaining splice site sequence [104]. Furthermore, there are *cis*-acting splicing regulatory elements (SREs) in both exons and introns that regulate splicing. The SREs are 6 to 8 nucleotides long and can positively (enhancers) or negatively (suppressors) affect splicing through recruiting trans-acting serine/arginine-rich (SR) proteins or heterogeneous nuclear ribonucleoproteins (hnRNPs), respectively.

Synonymous variants can either disrupt native splice sites, create de novo splice sites, activate cryptic splice sites, or affect SREs (those located in exons are called exonic splicing enhancers (ESEs) or exonic splicing silencers (ESSs)) and result in variable outcomes, including exon skipping and partial exon deletions [105]. Splicing dysregulation is arguably the best studied mechanism by which synonymous variants affect phenotypes and thus far has been implicated as the primary underlying mechanism for a majority of diseases caused by these variants [7]. A plethora of in silico tools have been developed for predicting the effects of genetic variants on splicing (Table 2).

**Table 2:** Select list of in silico tools for predicting mRNA splicing effects

Tool	Algorithm/prediction method	Input	Output	URL/comments	Ref
Splice site prediction tools					
GENSCAN	Motif-based, maximal dependence decomposition (MDD)	Sequences up to 1 million nucleotides can be analyzed	Predicted exons and/or peptides in the sequence	http://hollywood.mit.edu/GENSCAN.html A copy of the program is available on request	[106]
MaxEntScan	Motif-based, based on maximum entropy principle (MEP)	9 and 23 nucleotide long sequences for donor and acceptor site predictions respectively	Tool provides a score for the sequence indicating its strength as splice site	http://hollywood.mit.edu/burgelab/maxent/Xmaxentscan_scoreseq.html Offers multiple scoring models as options Perl scripts to run algorithm are available for download	[107]
Genesplicer	Motif-based, MDD with Markov models	Sequences of up to 200,000 nucleotides	Predicted acceptor and donor sites in the sequence with scores	https://www.cbcb.umd.edu/software/GeneSplicer/gene_spl.shtml Program is available for download	[108]
NetGene2	Machine-learning (ML) based, neural networks	One sequence between 200 and 100,000 nucleotides	Program provides predicted acceptor and donor sites with a confidence score	https://services.healthtech.dtu.dk/service.php?NetGene2-2.42 Program is available for download. The training dataset curated from NCBI GenBank included 65 human genes with 331 donor and acceptor splice sites	[109]
Spliceport	ML-based, support vector machine	Sequence of up to 30,000 nucleotides	Spliceport provides a list a predictions for donor and acceptor sites along with a score	https://spliceport.cbcb.umd.edu/SplicingAnalyser2.html Training dataset included a collection of 4000 pre-mRNA human RefSeq sequences	[110]
MMSplice	ML(deep learning)-based, modular neural networks	Predictive analysis of variants is performed on any exon with 50 and 13 nucleotides upstream and downstream respectively	Predicts effects of variants on exon skipping, splice site choice, splicing efficiency, and pathogenicity	Models are available in the Kipoi repository Trained on distinct large-scale genomics datasets. Please refer to Table 1 of citation for a detailed summary of trained modules and models	[111]
SRE prediction tools					
ESEfinder	Functional SELEX, PWM	Sequences of up to 5000 nucleotides	Tool provides predicted splice sites and SREs along with a score	http://krainer01.cshl.edu/cgi-bin/tools/ESE3/esefinder.cgi?process=home	[112]
EX-SKIP	Predictions are based on a ratio of ESE/ESSs identified from 5 different models including RECUE-ESE, FAS-ESSs	Two exonic sequences strictly in uppercase in FASTA format up to a total length of 4000 nucleotides	Program compares the ESE/ESS profile of a native and variant sequence and predicts the probability of exon skipping	https://ex-skip.img.cas.cz/	[113]
FAS-ESS	Experimental verification of random decanucleotide (10-nucleotides) sequences and weight matrices of aligned sequences	Single or multiple sequences in FASTA format. Length limit not specified	Program shows predicted ESS motifs. Users can search for hex2 or hex3 sets with relatively higher sensitivity and specificity respectively	http://hollywood.mit.edu/fas-ess/	[114]
ESRSeq	Experimental assessment of the SRE properties of all possible hexamer motifs	6-hexamer sequences overlapping the variants	Calculate the net ESRseq score. Net score change could indicate prediction direction	Scores of ESE/ESS hexamers are available as supplementary materials	[115]
Combination analysis tools					
SROOGLE	Predictions based on 9 different models including MEP, PWM, ESEFinder, RESCUE-ESE, FAS-ESS, etc	Target exon along with flanking introns	Provides predictions for both splice sites and SREs	http://sroogle.tau.ac.il/	[116]
ExonScan	Splice site predictions based on MEP and SRE predictions based on RESCUE-ESE and FAS-ESS	DNA sequence with exons with at least 20 bases upstream of the exon it can predict and 60 bases of downstream of the last	Provides predictions for both splice sites and SREs	http://hollywood.mit.edu/exonscan/	[114]

These tools can be broadly categorized as motif-based or ML- and DL-based algorithms [117]. Splice Site Finder-like (SSF-like, embedded in other platforms referenced below), Genscan [106], Genesplicer [108] and MaxEntScan (MES) [107] are examples of tools employing motif-based algorithms. Specifically, Spliceview and SSF-like employ position weight matrices (PWM) [118] to derive potential splice-site strength estimates for a sequence. Genscan uses a maximal dependence decomposition (MDD) model, which is a decision tree-based method that attempts to capture dependencies between both adjacent and non-adjacent positions. Genesplicer combines MDD with Markov models (MM) to capture additional dependencies between neighboring positions. MES uses maximum entropy principle (MEP) for modeling short sequence motifs found in splice sites while also accounting for higher-order dependencies between adjacent and non-adjacent positions. Some tools combine multiple algorithms or tools for their SS predictions. For example, Human Splicing Finder (HSF) [119] uses both PWM and algorithms from MES. On the other hand, SPiCE (Splicing Prediction in Consensus Elements) [120] uses logistic regression to combine MES and SSF-like tool predictions.

Increasingly, tools employing ML-based algorithms are being developed for SS prediction. NetGene2 [109], NNSplice [121], Alternative Splice Site Predictor (ASSP) [122], Spliceport [110], SpliceAI [123], MMSplice [111], and SpliceRover [124] are some examples in this category. Of these, NNSplice, NetGene2, and ASSP employ neural networks algorithms, while Spliceport employs a support vector machine algorithm. Similarly, tools based on other ML algorithms like random forest, naïve Bayes, and decision trees have been developed. More recently, DL technique-based tools employing deep/convoluted neural networks were developed, including SpliceAI, MMSplice, and SpliceRover [125]. These tools have exhibited promising results and are touted for freeing algorithms from the constraints of human intervention, while enabling the use of novel methods and parameters to identify splice sites and classify nucleotide variants [117].

Similar to splice site prediction, a variety of tools for predicting a genetic variant’s effect on SREs have been developed. ESEFinder [112], RESCUE-ESE [126], and FAS-ESS [114] were among the earliest developed SRE prediction tools. ESEFinder employs PWMs supported by functional SELEX ((Systematic Evolution of Ligands by EXponential enrichment) screen data to predict ESEs in the targeted sequence. RESCUE-ESE (Relative Enhancer and Silencer Classification by Unanimous Enrichment) employed a hybrid computational-experimental approach where putative ESEs were first predicted computationally and then experimentally verified by minigene assays. FAS-ESS employed experimental procedures (similar to functional SELEX) to screen random decanucleotide sequences and identify ESSs in the exon sequences. ESRSeq [115] and HEXplorer [127] are more recently developed tools for SRE prediction in exons. Of these, ESRseq analyzed the effects of all possible (4096) hexamer sequences on splicing using a minigene assay and categorized them as either ESEs or ESSs and assigned a score depending on the strength of effect. HEXplorer on the other hand employs a RESCUE-type in silico approach to categorize and assign scores for hexamer sequences. Additionally, tools like EX-SKIP [113] combine predictions of ESE/ESSs from multiple methods, including RESUCE-ESE and FAS-ESS and assign a score based on their relative density to indicate their ability to induce exon skipping.

A select list of tools performs predictions for both splice sites and SREs. For example, SROOGLE [116] provides predictions for both splice sites and SREs along with branch point sequences and PPT using 9 different algorithms. HSF provides splice site, SRE and BP predictions employing multiple algorithms. Similarly, ExonScan [114] provides splice site predictions using maximum entropy model and SRE predictions using RESCUE-ESE and FAS-ESS approaches.

The above discussed in silico tools were successfully used for the evaluation of splicing effects of genetic variants by multiple studies [12, 128–131]. Zhou et al. employed HSF and ESEFinder for the evaluation of naturally occurring synonymous variants in the *ATP7B* Gene [129]. Zhang et al. used SpliceSiteFinder-like, MaxEntScan, NNsplice, GeneSplicer, and HSF for the assessment of *F9* synonymous variants [130]. Overall, users have access to a large variety of tools. A majority of the tools provide scores indicating the strength of the splice site or SREs in a sequence of interest. A measure of change in score between native and variant sequences generally indicates the effect of the variant on splicing. While higher score changes generally indicate greater impact on splicing, there is no consensus on a threshold/cut-off score. Several studies were conducted to compare the performance of tools [132]; however, they are incomparable as they varied in both tools studied and test datasets and consequently differed in their conclusions. A recent comparative study with tools based on both motif-based and ML-based algorithms showed variable tool performances depending on the context of the test dataset [117]. Generally, predictions for variants located within consensus splice sites tend to be more accurate than for deep exonic variants [12]. For optimal use, the user needs to understand the features and limitations of individual tools. For example, the length of consensus SSs used in training varies between tools and not all tools were trained to identify noncanonical SSs (e.g., GC-AG and AT-AC). The presence and/or lack of tissue-specific splicing events in the training datasets could also influence predictions [117]. The type of input sequence required by tools, ability to perform batch analysis and the availability of source code will also influence tool choices. Use of a combination of tools predicting both SSs and SREs and employing different algorithms is recommended to overcome potential deficiencies of a single tool and is expected to improve predictive values [12, 132, 133].

## In silico tools for predicting the effect of synonymous variants on miRNA binding

miRNAs, short (17–22 nucleotides) single, non-coding RNAs, bind to the complementary sequences of target proteins and regulate their expression [134]. miRNA genes are located either in intergenic regions or within introns of protein coding genes. miRNA expression is cell-type and cell-state specific [135], and genetic variants can affect the gene regulation network. Numerous studies have demonstrated that single nucleotide variants within the miRNA or mRNA untranslated regions (UTR) can affect mRNA-miRNA interactions [136, 137], dysregulate protein expression by causing the gain or loss of miRNA binding sites within the gene’s coding sequence (CDS) [138], and may lead to disease pathogenesis [136]. In fact, recent studies estimated that nearly half of sSNVs can affect miRNA binding, disturb protein functions, and increase disease risk [15]. For example, a synonymous variant (c.313C > T) in *IRGM* disturbs the miR-196 binding site and dysregulates IRGM-dependent xenophagy in Crohn’s disease [14], and a synonymous variant (c.51C > T) in *BCL2L12*, identified in melanoma tumors, causes loss of the miR-671-5p binding site that stimulates protein expression [139].

The mechanism underlying miRNA association is complex and not fully understood, but the main interaction occurs via the 5′ seed region (nucleotides 2–8). Additional pairing at the 3′ end stabilizes the miRNA interaction [134]. Due to a non-perfect complementarity, miRNA can bind and regulate multiple genes through multiple binding sites either in the UTR or CDS regions [140].

As miRNAs regulate gene expression mainly by binding to their target sequence within 3′ untranslated region (3’UTR), most in silico tools have predominantly focused on miRNA target site predictions within the UTR [141]. Nevertheless, a few tools are currently available to identify miRNA target sites within the CDS and to study the effect of synonymous variants (Table 3). A large list of miRNA target prediction tools can be found on the Tools4miRs platform, which has amassed over 170 methods for broadly defined miRNA analysis (https://tools4mirs.org/). Here, we focused on tools that can be used to investigate genetic variants within the coding region.

**Table 3:** In silico tools for assessing effects of synonymous variants on miRNA binding

Tool	Algorithm/prediction method	Output/score	Year	URL	Ref
**TargetScan and Target Scan S**	Sequence alignment	Weighted context + + score (from -1 to 1). The scores with a lower negative value indicate a greater prediction of repression	2005	https://www.targetscan.org/vert_80/	[142, 143]
**MinoTar**	Sequence alignment and conservations scoring	Probability	2010	https://www.flyrnai.org/cgi-bin/DRSC_MinoTar.pl	[144]
		Conserved			
		Targeting			
**miRDB (MirTarget)**	Machine Learning (Support vector machine [SVM])	Target prediction scores between 50 and 100. A predicted target with prediction score > 80 is most likely to be real	2020	http://mirdb.org/	[145, 146]
**ComiR**	Machine learning (support vector machines)	Ranked vector of scores; therefore, each gene is associated with a reliability of being a target of the set of miRNAs given in input	2015 (updated in 2020 to include coding regions)	http://www.benoslab.pitt.edu/comir/help.html	[147, 148]
**Diana-microT**	microT-CDS algorithm	miTG score (from 0 to 1). The closer to 1, the greater the confidence	2009 (updated in 2013)	https://dianalab.e-ce.uth.gr/html/dianauniverse/index.php?r=microT_CDS	[149]
**Paccmit-CDS**	Ranking based on Markov model and sequence alignment	The predictions are ranked according to the *P*-value that the observed number of conserved and/or accessible seed matches would appear in the target sequence by chance	2015	https://paccmit.epfl.ch/	[150]
**miRanda**	Ranking based on seed match, conservation and free energy (G:U pairs allowed in the seed)	mirSVR score (< 0) is an estimate of the miRNA effect on the mRNA expression level. PhastCons score (0–1) measures the conservation of nucleotide positions across multiple vertebrates	2005 (updated in 2010)	https://cbio.mskcc.org/miRNA2003/miranda.html	[151, 152]
**PITA**	Ranking based on seed match, free energy, site accessibility and target-site abundance (G:U pairs allowed in the seed)	The predictions are ranked based on having a full match 7- or 8-mer seed and a conservation score of 0.9 or higher	2007	https://genie.weizmann.ac.il/pubs/mir07/mir07_prediction.html	[153]

TargetScan predicts biological targets of miRNAs by searching for the presence of conserved motifs (mer sites) within the gene that matches the miRNA seed region [142]. The online version of the tool is limited to the reference gene and is not specifically designed to predict miRNA binding site within the CDS. To analyze custom sequences, TargetScan provides a downloadable version of the code.

Another tool, MinoTar (miRNA ORF Target), predicts miRNA binding sites within the CDS by identifying highly conserved regulatory motifs [144]. However, the current version of the tool limits the prediction to reference sequences.

miRNA database (miRDB) searches for miRNA target sites through a support vector machines (SVMs) algorithm and is trained with high-throughput experimental datasets. The database can perform predictions in the CDS but is limited to native gene sequences. The tool allows for analyzing any customer mRNA sequence using the 3′ UTR region model [145]. In addition, the database was recently updated with cell-specific miRNA targets [146, 154].

ComiR (Combinatorial miRNA targeting) uses predictions from four common algorithms (PITA [153], miRanda [151], TargetScan [142], miRSVR [155]) and converts the results into a single probabilistic score using ensemble learning to predict whether a given mRNA is targeted by a set of miRNAs [147, 156]. This tool can accommodate custom mRNA sequences. The current version focuses on prediction within the 3′ UTR region, but the database may soon be upgraded to include CDS binding sites along with miRNA expression data. Preliminary studies have shown that information contained in the CDS significantly improves the accuracy of ComiR predictions [148].

DIANA-microT-CDS can identify miRNA targets in the 3′ untranslated region (3′ UTR) and in the CDS [149]. This algorithm uses miRNA-recognition elements (MREs) for the miRNA:mRNA base pairing. The software provides an automatic pipeline as well as plug-ins that allow the user to access the target prediction server and incorporate advanced miRNA analysis into custom pipelines.

Paccmit-CDS (Prediction of Accessible and/or Conserved MIcroRNA Targets) searches for potential microRNA targets within CDS by identifying conserved complementary motifs to the microRNA seed region and ranking them with respect to a random background that preserves both codon usage and amino acid sequence [150]. The tool presented on the website allows for evaluation of reference genes, but the program written in C +  + can be used to evaluate the effect of synonymous variants. Paccmit-CDS, TargetScan, and miRDB prediction tools have been recently used to evaluate for the effect of synonymous variants in ADAMTS13 [157].

MiRanda, which is accessible online, allows searches for miRNA binding sites within the 3′ UTR region of specific genes, by inputting gene names. Installing the miRanda package allows for the detection of potential microRNA target sites in genomic sequences and can be used to evaluate the effect of synonymous variants [151, 152].

The online miRNA prediction tool, PITA, can process UTR sequences. While it is not designed to study miRNA binding sites within the CDS, it was previously used in concert with miRanda to identify miRNA target sites, encompassing the C51T variant site in BCL2L12 [139].

For validation of miRNA binding sites within the protein coding region, these prediction software require input of the gene sequence, which is then aligned with miRNA sequences derived from miRbase [158]. By comparing the outcome of the WT sequence, which is defined by a list of predicted miRNAs and with associated scores generated by specific prediction tools, with the list of miRNAs predicted to bind the variant sequence, the gain or loss of miRNA binding can be determined.

The main limitations of some current prediction algorithms are that they are based on conservation and are not fully adapted for processing the CDS. Many tools neglect consideration of cell-type specific miRNA expression levels, do not consider target site availabilities due to protein folding, and limit the analysis to a reference gene sequence. Since mRNA-miRNA association is based on non-perfect complementarity, the outcome data contains hundreds of predicted miRNAs, and it is advisable to validate miRNA predictions by comparing the output data from three or more prediction tools. As synonymous variant prediction outcomes within the CDS have not been extensively validated, and variants that have been experimentally assessed do not always support the prediction algorithms [159], it is difficult to recommend a specific tool that is best for forming SNV miRNA predictions. Nevertheless, many tools have recently evolved to include CDS analysis and the development of more robust bioinformatic and experimental methods to evaluate miRNA alterations by synonymous variants remains an ongoing pursuit.

## In silico tools for predicting pathogenicity of synonymous variants

As more synonymous variants are being implemented in the development of genetic therapies and drugs, the creation of more powerful tools to predict functional synonymous variants has become even more important. Many discovered synonymous variants have been linked to increased risks for developing diseases and cancers [9]. For example, synonymous variants have been found to underlie Hemophilia [77, 160] and in cancer, about 6–8% of pathogenic single nucleotide substitutions identify as synonymous variants [161]. As a result, there is growing interest in the development of in silico tools that can reliably predict the pathogenicity of synonymous variants.

Currently, methods to predict rare coding variants, mostly targeting pathogenic missense variants, have proven to be quite effective, such as REVEL [162] and CADD [24]. However, progress towards predicting pathogenic synonymous variants remains far behind. While creating pathogenic synonymous variant prediction tools is complicated and challenging, recent progress towards this objective has come on the heels of advancements in ML platforms and greater insight on the importance of a variety of sequence properties in influencing disease. mRNA metrics and protein-associated variables, such as amino acid conservation, have been considered in algorithms to predict pathogenicity [21, 163]. In addition, generation of robust prediction tools is highly dependent on the availability of disease-associated genetic data that can be used to train ML systems. Numerous data sets have been curated with information on disease-related variants, such as Human Gene mutation database (HGMD) [164] and VariSNP [165], and there are numerous resources for curating neutral synonymous variants, including the 1000 Genomes Project (1000G) [166, 167]. But, while these are the most extensive datasets and have been used to train ML prediction tools, these datasets require further improvements. Unfortunately, as many have noted [168], there are inconsistencies in characterizations, nomenclature, and disease annotations in these databases, which have encouraged many recent efforts to correct these annotation flaws [169]. However, these factors have made it exceedingly difficult to generate accurate disease predictions.

Nevertheless, many ML tools based on supervised algorithms, such as random forests (RFs), deep neural networks, or support vector machine (SVMs), have been generated with reasonable proficiencies at predicting pathogenic synonymous variants. Some examples of such tools include SilVA (Silent Variant Analyzer) [22], DDIG-SN (Detecting Disease-causing Genetic SynoNymous variants) [23], IDSV (Identification of Deleterious Synonymous Variants) [163], and TraP (Transcript-inferred Pathogenicity) [170]. Each of these tools utilize a different assortment of features to predict pathogenicity of synonymous variants, but the most common implemented features include conservation, splicing, and RNA folding metrics. Most of these tools require a list of variants, formatted as VCF or tag-like files, and will rank synonymous variants based on their predicted pathogenicity. While it seems unreasonable to compare the accuracies of prediction tools due to the lack of an ideal standardized testing set, Zeng and colleagues found that when tested with a mock dataset, SilVA, DDIG-SN, and TraP were highly correlated in their predictive capacities but were not effective at large-scale variant predictions [171].

Ultimately, improvements in variant predictors will only occur with enhancements to genetic data sets. usDSM (Deleterious Synonymous Mutation Prediction using Undersampling Scheme) [172] and synVep (Synonymous Variant Effect Predictor) [21] are newer tools that have demonstrated improved proficiencies by implementing undersampling methods and positive-unlabeled learning, respectively, to circumvent the lack of robust training sets. In addition, concerted efforts have been made to create artificial datasets to train prediction models [171]. Alternatively, transitioning from a supervised ML system to unsupervised or semi-supervised methodologies may help to overcome the scarcity of available data. These methods are advantageous as they eliminate biases by removing the need for predefined labels like “pathogenic or benign” in training sets. One example of an unsupervised prediction tool is ParsSNP [173], which has outperformed existing tools in identifying driver mutations of cancer. However, specific application of unsupervised methods for synonymous variant prediction has not been adopted.

## Importance of in vitro validation of in silico tool predictions in synonymous variant research

While computational tools for evaluating synonymous variants have improved significantly in recent years, in silico tools are still fundamentally imperfect systems. In many cases, predicted disease variants do not mirror the actual biological outcomes due to unknown biological complexities or deficiencies in the number of reliable and comprehensive genomic data sets. Therefore, it is increasingly important that in silico tool predictions be performed by multiple prediction tools with a variety of algorithms and parameters and validated through in vitro experiments. Currently, examples of experimentally corroborated synonymous variants are still quite low, which can be partially attributed to the necessity for more sensitive, standardized experimental assays. Detected protein or RNA alterations are usually significant, but small in magnitude. Many seminal works began as studies that leveraged the power of synonymous variant prediction tools to identify potential candidates and followed up these findings with experimental confirmation (see Table 4 for examples from highly cited studies that employed a combination of in silico and in vitro experiments to effectively investigate sSNV mechanisms). For a thorough review of experimental methods and discussion of studies that have investigated synonymous variants, we recommend reviewing Chapter 7 of a recently published book on *Single Nucleotide Polymorphisms* [174]. With the incessant rise in accumulations of genetic data and improving landscape of computational tools, the number of functional synonymous variants should dramatically increase over the next decade.

**Table 4:** Examples of studies that effectively used prediction tools to study disease-causing synonymous variants

Disease association	Variant	Prediction tool	Description	Ref
Crohn’s disease	***IRGM*** (c.313C > T)	SnipMir, RegRNA, and Patrocles (miRNA)	Synonymous variant predicted to delete a miRNA binding site, leading to increased risk for Crohn’s disease (validated to be the causal mechanism through experiments assessing IRGM regulation)	[175]
Cystic fibrosis	***ΔF508 CFTR*** (c.1520_1522delTCT)	mFold (mRNA structure)	Synonymous site within the ΔF508 CFTR predicted to alter mRNA structure and stability and found to responsible for altered expression of the mutant protein	[18, 176]
Hemophilia B	***FIX*** [Factor IX] (c.459G > A)	mFold, Kinefold, NUPACK (mRNA structure), RSCU, CAI (codon usage indices)	mRNA structure prediction tools indicated a moderate reduction in mRNA stability, which coincided with diminished FIX expression through decreased translational speed	[77]
Hereditary cardiac arrhythmia	***hERG*** (codon-modified)	RNAfold (mRNA structure)	Codon modified hERG was predicted to have increased mRNA stability, resulting in altered translation of the ion channel	[19]
Pain sensitivity	***COMT*** (3 haplotypes with synonymous variations [c.198A > G, c.186C > T, c.408C > G])	mFold (mRNA structure)	COMT haplotype with predicted highest mRNA stability correlated with the lowest activity and expression levels. Other haplotypes with different thermodynamic stabilities elicited different pain sensitivities	[147, 177]
Phenylketonuria	***PAH*** (c.30C > G)	ESE Finder 3.0 (splicing)	An exonic splicing silencer was identified through splicing predictions and validated experimentally to be the main mechanism underlying the PKA-causing variant	[178]
Tuberculosis	***mabA*** (c.609G > A)	GENETYX-MAC (promoter prediction)	Synonymous variant predicted to cause the formation of an alternative promoter site, next to the mutation position, which was validated and found to increase transcription of inhA, leading to increased isoniazid resistance	[155, 179]

## Concluding remarks and future perspectives

While overlooked in the past, synonymous variants are now recognized for their numerous functional effects and contribution to diseases. While this change in perspective was certainly precipitated by the rapid expansion of genetic testing and improvements in sequencing technologies, it must also be ascribed to recent significant advancements in bioinformatic AI and ML platforms. As highlighted in this review, in silico tools, especially those rooted in machine-learning algorithms, have been used to enhance our understanding of mechanisms underlying synonymous variants, while giving rise to additional inventive ideas, such as leveraging synonymous variants in genomic engineering strategies (e.g., codon optimization) to develop therapeutics [180]. In addition, the identification of recurrent disease mechanisms among synonymous variants, such as splicing or disrupted mRNA structure, has facilitated the discovery of new synonymous variants in other disease states, such as cancers [159]. The extended application of these technologies will be dependent on whether continued progress can be made in developing accurate synonymous variant computational predictors as these tools represent the most efficient means to process large-scale variant datasets. In the short term, the shortage of reliable genetic datasets on synonymous variants remains a significant obstacle for their rapid improvement, but as sequencing continues to become affordable and commonly used, this issue may be resolved naturally over time.

Thus, in the near future, promising improvements in these prediction tools may originate from enhanced understanding of codon, RNA, and sequence properties that correlate with functional synonymous variants. Future studies will need to address many outstanding questions in this field, including determining whether an array of sequence features can accurately discriminate functional or pathogenic synonymous variants. In addition, it will be important to develop refined models, specifically intended for synonymous variants, as many existing methods rely on adapting generic tools for synonymous variant assessment. This is suboptimal, as certain tools may place greater emphasis on particular variables and may not be able to sensitively detect functional variants. Fortunately, our understanding of biological relationships between codon usage, mRNA structure, and other protein sequence features continues to improve, and once intractable questions, such as how synonymous variants can alter the specific activity of proteins, have now been described [181]. The incorporation of these new variables into the design of in silico tools and the expanding use of these tools by the broad research community will only help to expedite novel discoveries in synonymous variant research.

## Supplementary Information

**Additional file 1.** Review history.
