# Supplementary material for: In silico methods for predicting functional synonymous variants
Source: Genome Biol. 2023 May 22;24:126. doi: 10.1186/s13059-023-02966-1 (PMC10204308; doi:10.1186/s13059-023-02966-1)
Supplement: Supplementary file 1 — Additional file 1. Review history. [file 13059_2023_2966_MOESM1_ESM.docx]

Review History

**First round of review**

**Reviewer 1**

**Comments to author:**

Synonymous SNVs (sSNVs) are an important category of genetic variations, which were often assumed to be "silent" in most genomic studies. In this review paper, Lin and coauthors summarized the current findings in predicting functional synonymous variants. The authors categorized the mechanisms of the functional impact of sSNV into 4 classes: CUB, RNA structure/stability, splicing and miRNA binding. Detailed molecular mechanism, available prediction software and a supportive example are provided for each of the classes. The materials are clearly presented, and easy to read.

Here are some major comments:

1. In line 120-121, the authors mentioned that "the realized structure is likely one that requires the least amount of folding energy". The wording here might be misleading, as RNA don't require energy to fold but rather release energy when folding. I think what the authors want to say is "the realized structure is likely one that possess the lowest free energy".

2. The authors spent a lot of efforts summarizing the different tools that can be used to predict mRNA structure and stability. The output for most of those tools should be a list of predicted mRNA structures with different free energy changes (deltaG). However, it is unclear to me how to use those values to infer the variant impact. Maybe check the free energy change with and without the variant, but what is a good deltaG difference to indicate the variant is impactful? The workflow shown in Figure 1 is great, but it is never referred to in the main text, and more details should be added.

3. The software summarizing table is very useful. But additional information could improve the table. It would be great to include the input and output for each software (some of them are scattered in the table). For ML algorithms, it would be great to include which dataset is used for training.

4. Many methods are summarized in the manuscript. Missing, however, is any recommendation to help us prioritize some of them over the others?

5. Lines 103: The authors described the tools and a few example variants but without many details on how the variants might be functionally important and lead to modified or unstable mRNA structures. Readers would expect deeper descriptions and discussions on expected energy with wildtype, energy of modified mRNA with sSNV, and delta energy between them. As mentioned before, Table 1 categorized two prediction algorithms - Free Energy Minimization, Psedoknots, ... but these algorithms never described, compared, or discussed. It was not well described how the tools and the algorithms should be used effectively although the authors claimed line 59-61. Similar issue found again in next section - miRNA binding.

6. Line 249. This section is too long and would benefit from careful editing.

7. Table 3 appears to have an empty column (#7).

8. Line 254: How are SNP different from SNV? It many be better to use the same term SNV across manuscript. Perhaps, it would be a good to include "Glossary box" to describe several important terms.

9. Line 342-344: It seems reference(s) missing.

10. Line 346: 1KG is unrelated to disease phenotypes or variants. If 1KG is used for ML training, it may be used for negative control dataset not as disease variants data. This must be clearly described. If included, the proper paper should be used for reference instead of news (2008) as the project published in 2015.There are potentially more disease related associated db such as ClinVar, TCGA, etc.

11. Line 332: This section is well covered and written, but lacks information relevant to sSNVs.

12. Line 348-351 Please explain why it needs improvement and if possible, suggest how.

13. Line 376: This section might be expanded as it does describe how the sSNVs can be effectively studied. Indeed, missing are details of In-vitro validation methods the title specifically call attention to.

14. Line 381 said it is low not 0. Please described how the studies validated sSNVs experimentally with references. Similarly, Table 4 can be expanded, there are only 10 studies that effectively investigated sSNVs? How the 10 studies selected based on which criteria?

Here are some minor comments:

1. The term "codon pair" might not be familiar to people outside the field. It probably meant 2 consecutive codons in the mRNA. A brief explanation would help.

2. Page 4 line 80, "TAI calculator" should be "tAI calculator".

3. A lot of numbers are listed in line 163 to line 169, but no references are cited.

4. The resolution for Figure 1 is very low.

5. Lines 59-61. These sentences are useful. But can the authors give additional insights on how the tools can be used effectively.

The prediction of variant impact of sSNVs is an important question for genomic research. Parts of the manuscript are clearly written and provide useful information regarding this topic. But the writing and depth of analysis is uneven and many improvements can be made. I recommend this manuscript for a major revision.

**Reviewer 2**

**Comments to author:**

This review is useful and pretty comprehensive. It is very frustrating to read because it is wordy and filled with flowery language and unnecessary adjectives and adverbs. This diminishes the value to a reader. With appropriate editing and 20% shrinkage (with no loss of content), it will be a good manuscript.

Minor notes

* You may want to mention the utility of 3D modeling of RNA (like Eterna and other tools) for understanding the impact of nonsynon mutations

* In the review of general methods for pathogenesis, you should include at least a mention of the general purpose tools like CADD and REVEL and that class of tools.

Example edits that a copy editor should do

* what does "are the underpinnings" mean? Use more straightforward language

"existing and recently developed" = everything = no content = delete

"spurred greater advantages" what does this mean? Simplify

You do not mean "variation in the genetic code" on line 22! The genetic code maps codons to amino acids, and although there are interesting variations, I think you are talking about DNA sequence variation and not variation in the genetic code!

line 24/25 = confuses individuality and differences = need to make up your mind

spanning from = including

delete unequivocal on line 33

please do not discuss dogma in 43-44 just delete that whole phrase

line 51: codon usage and GS content is NOT a protein characteristic! Those are for RNA or DNA

You get the idea, this is just my reaction to the first few pages.

**Authors Response**

**Point-by-point responses to the reviewers’ comments:**

**Referee 1:**

Synonymous SNVs (sSNVs) are an important category of genetic variations, which were often assumed to be "silent" in most genomic studies. In this review paper, Lin and coauthors summarized the current findings in predicting functional synonymous variants. The authors categorized the mechanisms of the functional impact of sSNV into 4 classes: CUB, RNA structure/stability, splicing and miRNA binding. Detailed molecular mechanism, available prediction software and a supportive example are provided for each of the classes. The materials are clearly presented, and easy to read.

*Response: We thank the reviewer for this positive assessment of our manuscript and*

*for providing thoughtful suggestions and recommendations for improving our*

*manuscript.*

Here are some major comments:

1. In line 120-121, the authors mentioned that "the realized structure is likely one that requires the least amount of folding energy". The wording here might be misleading, as RNA don't require energy to fold but rather release energy when folding. I think what the authors want to say is "the realized structure is likely one that possess the lowest free energy".

*Response: We thank the reviewer for pointing out this misleading sentence. We have*

*corrected the sentence to read: “the realized structure is one that possesses the lowest*

*free energy” on pg. 5, lines 114-116.*

2. The authors spent a lot of efforts summarizing the different tools that can be used to predict mRNA structure and stability. The output for most of those tools should be a list of predicted mRNA structures with different free energy changes (deltaG). However, it is unclear to me how to use those values to infer the variant impact. Maybe check the free energy change with and without the variant, but what is a good deltaG difference to indicate the variant is impactful? The workflow shown in Figure 1 is great, but it is never referred to in the main text, and more details should be added.

*Response: We thank the reviewer for these suggestions. We have enhanced the*

*discussion on how to effectively implement mRNA prediction tools for synonymous*

*variant prediction, including a discussion of free energy changes. Please see the*

*revised mRNA structure/stability section and specifically, pgs. 6-7, lines 135-147, in*

*which we additionally reference the workflow figure.*

3. The software summarizing table is very useful. But additional information could improve the table. It would be great to include the input and output for each software (some of them are scattered in the table). For ML algorithms, it would be great to include which dataset is used for training.

*Response: We thank the reviewer for this suggestion. We have modified Tables 1 and*

*2 to include input and output columns, and added additional comments to Table 1,*

*regarding training sets for ML tools (ex. Table 1; SPOT-RNA - trained with bpRNA*

*[101] and PDB [102] databases). For the miRNA section and Table 3, outputs are*

*described directly in the table, while inputs are described in the text (pg. 19, lines 343-*

*347) as miRNA prediction tools have generally the same input requirements.*

4. Many methods are summarized in the manuscript. Missing, however, is any recommendation to help us prioritize some of them over the others?

*Response: We thank the reviewer for identifying this point for further discussion. It is*

*our viewpoint that it is very difficult to prioritize a particular tool over others due to the*

*varying parameters and algorithms implemented by these tools. Some tools may be*

*more effective at predicting the effects of variants for a specific gene but may be less
effective for others. Each tool involves specific variables, such as inputs (e.g,*

*nucleotide lengths) that can affect the effectiveness of their outputs. We have*

*expanded upon many of these features and have elaborated further upon in the text*

*and accompanying tables. Our recommendation is to use multiple tools to evaluate*

*synonymous variant effects and to validate the predictions with experimental methods,*

*which is supported by studies that have compared prediction tools (ex. refs 107, 133).*

*We have now ensured that this recommendation along with supported reasoning are*

*highlighted in various sections of the review (see pgs. 8-9, lines 186-195; pgs. 14-15,*

*lines 266-279; pgs. 19-20, lines 348-359; pg 24, lines 410-415).*

5. Lines 103: The authors described the tools and a few example variants but without many details on how the variants might be functionally important and lead to modified or unstable mRNA structures. Readers would expect deeper descriptions and discussions on expected energy with wildtype, energy of modified mRNA with sSNV, and delta energy between them. As mentioned before, Table 1 categorized two prediction algorithms - Free Energy Minimization, Psedoknots, ... but these algorithms never described, compared, or discussed. It was not well described how the tools and the algorithms should be used effectively although the authors claimed line 59-61. Similar issue found again in next section - miRNA binding.

*Response: We thank the reviewer for this suggestion. As recommended, we have*

*expanded the discussion of the impact of synonymous variants on RNA structure and*

*miRNA binding. Please see the revised mRNA stability/structure section, specifically*

*pgs. 6-7, lines 135-159, and for the miRNA section, see changes to section highlighting*

*effects of synonymous variants on miRNA (pg 17) and for algorithms/tools (pgs. 19-20,*

*lines 343-359).*

6. Line 249. This section is too long and would benefit from careful editing.

*Response: We have revised and reduced the section (see revisions made in pgs. 17-*

*20).*

7. Table 3 appears to have an empty column (#7).

*Response: We have removed the empty column within Table 3.*

8. Line 254: How are SNP different from SNV? It many be better to use the same term SNV across manuscript. Perhaps, it would be a good to include "Glossary box" to describe several important terms.

*Response: We have unified the SNP/SNV term throughout the manuscript.*

9. Line 342-344: It seems reference(s) missing.

*Response: We have added the references to the corresponding lines: “mRNA metrics and protein-associated variables, such as amino acid conservation have been considered in algorithms to predict pathogenicity [166, 167]”.*

10. Line 346: 1KG is unrelated to disease phenotypes or variants. If 1KG is used for ML training, it may be used for negative control dataset not as disease variants data. This must be clearly described. If included, the proper paper should be used for reference instead of news (2008) as the project published in 2015.There are potentially more disease related associated db such as ClinVar, TCGA, etc.

*Response: We thank the reviewer for identifying the inaccuracy in the database descriptions. We have corrected the discussion of the 1KG database and its associated references. See Pg. 22, lines pgs. 376-381.*

11. Line 332: This section is well covered and written, but lacks information relevant to sSNVs.

*Response: We thank the reviewer for this comment. The overall landscape of predicting pathogenic sSNVs is far behind that of other pathogenic variant predictors. We have modified the sSNV section to enhance the discussion and to demonstrate the recent progress in the field. See pgs. 22-24.*

12. Line 348-351 Please explain why it needs improvement and if possible, suggest how.

*Response: We thank the reviewer for this recommendation. We have modified this section, see pgs. 22-23, lines 381-385.*

13. Line 376: This section might be expanded as it does describe how the sSNVs can be effectively studied. Indeed, missing are details of In-vitro validation methods the title specifically call attention to.

14. Line 381 said it is low not 0. Please described how the studies validated sSNVs experimentally with references. Similarly, Table 4 can be expanded, there are only 10 studies that effectively investigated sSNVs? How the 10 studies selected based on which criteria?

*Response to Comments 13 and 14: We thank the reviewer for these suggestions. Although we do want to call attention to the use of in vitro validation to support computational predictions, we would like to focus the attention of this review mainly on computational tools and their use in evaluating synonymous variants. The studies presented in Table 4 were chosen because they are highly cited studies that have specifically used the discussed computational approaches to identify a potential sSNV mechanism and then followed-up the study with investigating the computational prediction by thorough in vitro validation. For insights into experimental methods for evaluating synonymous variants, we have written a book chapter, published in 2022, which provides an expanded discussion of sensitive experimental methods, and which additionally includes examples of how they were used in many sSNV studies. We have added a referral to this book discussion in the corresponding section (see pg. 24, lines 420-422).*

Here are some minor comments:

1. The term "codon pair" might not be familiar to people outside the field. It probably meant 2 consecutive codons in the mRNA. A brief explanation would help.

2. Page 4 line 80, "TAI calculator" should be "tAI calculator".

3. A lot of numbers are listed in line 163 to line 169, but no references are cited.

4. The resolution for Figure 1 is very low.

5. Lines 59-61. These sentences are useful. But can the authors give additional insights on how the tools can be used effectively.

*Response: We thank the reviewer for providing us with these minor comments. We have addressed all the minor comments listed above in their appropriate section/lines.*

The prediction of variant impact of sSNVs is an important question for genomic research. Parts of the manuscript are clearly written and provide useful information regarding this topic. But the writing and depth of analysis is uneven and many improvements can be made. I recommend this manuscript for a major revision.

*Response: We thank the reviewer for the careful and thoughtful review of the*

*manuscript. As recommended, we have revised and enhanced the depth of analysis*

*throughout the manuscript.*

**Referee 2**

This review is useful and pretty comprehensive. It is very frustrating to read because it is wordy and filled with flowery language and unnecessary adjectives and adverbs. This diminishes the value to a reader. With appropriate editing and 20% shrinkage (with no loss of content), it will be a good manuscript.

*Response: We thank the reviewer for this positive assessment of our manuscript. We revised the manuscript by shrinking some sections while retaining content and eliminated wordiness and language that detracts from the scientific discussions.*

Minor notes

* You may want to mention the utility of 3D modeling of RNA (like Eterna and other tools) for understanding the impact of nonsynon mutations

*Response: We thank the reviewer for this recommendation. We have added a discussion of 3D modeling tools for evaluating RNA structure (see pg. 8, lines 171-185).*

* In the review of general methods for pathogenesis, you should include at least a mention of the general purpose tools like CADD and REVEL and that class of tools.

*Response: We thank the reviewer for this suggestion. We have added a mention of these general class of pathogenic variant predictors (see pg. 22, lines 370-372).*

**Second round of review**

**Reviewer 2**

*I thank the authors for responding to my comments and for making the manuscript much easier to read. It is a very nice contribution.*
